# Gut Microbiota in Patients Receiving Dialysis: A Review

**DOI:** 10.3390/pathogens13090801

**Published:** 2024-09-15

**Authors:** Xintian Lim, Lijin Ooi, Uzhe Ding, Henry H. L. Wu, Rajkumar Chinnadurai

**Affiliations:** 1 Department of Renal Medicine, Northern Care Alliance NHS Foundation Trust, Salford M6 8HD, UK; xintian.lim@nca.nhs.uk (X.L.); lijin.ooi@nca.nhs.uk (L.O.); uzhe.ding@nca.nhs.uk (U.D.); 2 Renal Research Laboratory, Kolling Institute of Medical Research, Royal North Shore Hospital, The University of Sydney, Sydney, NSW 2065, Australia; hon.wu@sydney.edu.au; 3 Faculty of Biology, Medicine & Health, The University of Manchester, Manchester M1 7HR, UK

**Keywords:** gut microbiota, chronic kidney disease, end-stage kidney disease, dialysis

## Abstract

The human gut microbiota constitutes a complex community of microorganisms residing within the gastrointestinal tract, encompassing a vast array of species that play crucial roles in health and disease. The disease processes involved in chronic kidney disease (CKD) and end-stage kidney disease (ESKD) are now increasingly established to result in dysregulation of gut microbiota composition and function. Gut microbiota dysbiosis has been associated with poor clinical outcomes and all-cause mortality in patients with ESKD, particularly individuals receiving dialysis. Prior studies highlighted various factors that affect gut microbiota dysbiosis in CKD and ESKD. These include, but are not limited to, uraemic toxin accumulation, chronic inflammation, immune dysfunction, medications, and dietary restrictions and nutritional status. There is a lack of studies at present that focus on the evaluation of gut microbiota dysbiosis in the context of dialysis. Knowledge on gut microbiota changes in this context is important for determining their impact on dialysis-specific and overall outcomes for this patient cohort. More importantly, evaluating gut microbiota composition can provide information into potential targets for therapeutic intervention. Identification of specific microbial signatures may result in further development of personalised treatments to improve patient outcomes and mitigate complications during dialysis. Optimising gut microbiota through various therapeutic approaches, including dietary adjustments, probiotics, prebiotics, medications, and faecal transplantation, have previously demonstrated potential in multiple medical conditions. It remains to be seen whether these therapeutic approaches are effective within the dialysis setting. Our review aims to evaluate evidence relating to alterations in the gut microbiota of patients undergoing dialysis. A growing body of evidence pointing to the complex yet significant relationship which surrounds gut microbiota and kidney health emphasises the importance of gut microbial balance to improve outcomes for individuals receiving dialysis.

## 1. Introduction

The human gut microbiota constitutes a complex community of microorganisms residing within the gastrointestinal tract, encompassing a vast array of species that play crucial roles in maintaining human health. Containing approximately 10^13^ to 10^14^ microorganisms and spanning 500 to 1000 bacterial species, this ecosystem influences various physiological processes [1]. The balance and diversity of these microbial populations are integral to the host’s overall well-being, contributing significantly to nutrient absorption, immune system modulation, and protection against pathogens [2,3]. Lower bacterial diversity has been repeatedly observed in individuals with inflammatory and cardiovascular diseases [4].

Dialysis is a form of kidney replacement therapy (KRT) that acts as a life-sustaining treatment for individuals with end-stage kidney disease (ESKD), a condition characterised by irreversible kidney failure. This therapy effectively substitutes the essential functions of the kidneys, such as the filtration of blood and removal of waste products and excess fluids. The two following primary modalities of KRT exist: (1) haemodialysis (HD), which involves filtering blood through a dialyser outside the body, and (2) peritoneal dialysis (PD), wherein the peritoneal membrane serves as a natural filter within the abdomen. Both methods aim to preserve the body’s homeostasis, vital for patient survival and overall health.

The rising incidence of chronic kidney disease (CKD) and its progression to ESKD have led to heightened mortality rates and a considerable socioeconomic burden. These conditions are recognised for causing significant disturbances in gut microbiota composition and function, resulting from factors such as uremic toxin accumulation, chronic inflammation, immune dysfunction, medications, and dietary restrictions and nutritional status [5,6,7]. Importantly, dysbiosis, or imbalance in the gut microbiota, is strongly correlated with all-cause mortality in dialysis patients [8]. Nevertheless, there is a relative paucity of studies focusing specifically on trends among dialysis patients. Understanding these changes is crucial for elucidating their impacts on patient outcomes, including complications relating to kidney disease and overall health. 

Investigating the shifts in gut microbiota composition during dialysis can provide insights into potential therapeutic targets to enhance patient management and outcomes. By identifying specific microbial signatures associated with dialysis, clinicians and researchers can develop personalised interventions to mitigate complications and improve the overall well-being of these patients. Optimising gut microbiota through various therapeutic approaches, including dietary adjustments, probiotics, prebiotics, medications, and faecal transplantation, holds the potential to alleviate these complications [9]. This review aims to explore the alterations in the gut microbiota of patients undergoing dialysis and to contribute to the growing body of knowledge surrounding the intricate relationship between gut microbiota and kidney health, emphasising the importance of maintaining gut microbial balance in improving outcomes for individuals undergoing dialysis.

## 2. Alterations in Gut Microbiota in CKD and Dialysis Patients

In a healthy human body, different segments of the gastrointestinal tract host diverse communities of microorganisms. The composition of gut microbiota varies based on factors such as age, geography, health condition, lifestyle, and genetics. Despite these variations, research indicates that the gut microbiota typically maintains stability in healthy individuals and is characterised by six major phyla: Firmicutes, Bacteroidetes, Actinobacteria, Proteobacteria, Fusobacteria, and Verrucomicrobia. Among these, Firmicutes, Bacteroidetes, Actinobacteria, and Proteobacteria are the most prevalent [10,11,12,13]. At the genus level, key constituents of the intestinal flora include *Bacteroides*, *Prevotella*, *Ruminococcus*, *Megamonas*, and *Faecalibacterium* [14].

This intricate microbial community interacts dynamically with other organs and body systems, playing a vital role in maintaining overall health and immunity. A balanced gut microbiota is crucial for nutrient metabolism, immune system modulation, preservation of barrier function, and promotion of metabolic health [15,16]. The gut microbiota aids in digestion, synthesising vitamins, and fermenting dietary fibres into short-chain fatty acids (SCFAs), essential for colon health. It reinforces intestinal integrity by simulating mucus production and the formation of tight junction proteins, which help prevent harmful substances from entering the bloodstream. Moreover, these microorganisms influence metabolic processes and energy homeostasis, impacting conditions such as obesity, type 2 diabetes mellitus, hypertension, and heart disease [16,17,18,19].

Dysbiosis of the gut microbiota manifests in patients with CKD and those undergoing dialysis [10,15,20]. According to a systematic review conducted by Voroneanu et al., microbial diversity tends to decrease progressively from the early to advanced stages of CKD [21]. Generally, CKD patients experience a reduction in anti-inflammatory microbes such as *Roseburia*, *Prevotella*, and *Bacteroides*, alongside an enrichment in pro-inflammatory microbes like Proteobacteria and Actinobacteria [21]. Another study highlighted that CKD patients exhibit higher relative abundances of *Klebsiella*, *Escherichia coli* (*E. coli*), Enterobacteriaceae, Lachnospiraceae, and *Fusobacterium* compared to their healthy counterparts [22].

A study by Wang et al. revealed that, as CKD progresses, there is a marked increase in the abundance of four pathogenic bacteria: *Citrobacter freundii*, *Citrobacter werkmanii*, *Flavonifractor plautii*, and *Anaerostipes caccae* [23]. The study also reported a decline in 14 species of SCFA-producing bacteria, which are essential for maintaining gut health [23]. Functionally, gut dysbiosis in CKD was closely connected to disruptions in arginine and proline metabolism, amino acid metabolism, glutathione metabolism, and the biosynthesis of ubiquinone and other terpenoid quinones [23]. These metabolic disturbances contribute to the accumulation of uremic toxins, trigger inflammatory responses, and amplify oxidative stress [23]. Voroneanu et al.’s systematic review concluded that the abundance of *Roseburia*, a beneficial gut bacterium, decreases as CKD advances [21]. Additionally, Gao et al. reported a progressive increase in *Bifidobacterium* levels during the later stages of CKD, whilst *Lactobacillus* levels showed a notable decline [14]. Research by Chen et al. further identified gradual increases in the relative abundances of *Klebsiella pneumoniae*, *Streptococcus criceti*, and *Haemophilus parainfluenzae* as CKD progressed, compared to in healthy individuals [24]. 

Metabolites produced by the microbiome, such as SCFAs, uremic toxins, and lipopolysaccharides, play crucial roles in maintaining human body homeostasis and are intricately linked to the development and progression of CKD [25]. Wang et al. observed an enrichment in urea cycle products in the serum of CKD patients, including creatinine, citrulline, and argininosuccinic acid [23]. Conversely, these urea cycle metabolites were depleted in faecal matter, such as N2-succinyl-L-ornithine, ornithine, and argininosuccinic acid. This imbalance may exacerbate CKD progression [23]. In relation, Chen et al. described an incremental increase in the levels of four faecal metabolites across various stages of CKD, including S-adenosylhomocysteine, propionic acid, myristic acid, and L-carnitine [24]. These findings suggest that gut metabolites could serve as valuable biomarkers for assessing or predicting the severity of CKD [24]. 

Patients undergoing dialysis experience reduced microbial diversity, marked phylum-level shifts, an increase in uraemic toxin producers, and a decline in SCFA-producing bacteria. Recent studies indicated no significant difference in α-diversity between individuals with normal kidney function and those on dialysis, but a notable disparity in β-diversity between these cohorts (α-diversity is a measure of microbiome diversity applicable to a single sample; β-diversity is a measure of the similarity or dissimilarity of two communities) [25,26,27]. In both the dialysis and healthy groups, Firmicutes consistently dominate as the primary phylum, followed by Bacteroidetes [28].

In comparison to healthy individuals, Firmicutes, Actinobacteria, and Proteobacteria are enriched in HD patients, whilst Bacteroidetes and Verrumicrobia are diminished [26,29]. Specifically, Proteobacteria have been found to exhibit the highest relative abundance in the HD group, according to Gao et al. [14]. At the genus level, HD patients typically harbour *Bacteroides*, *Blautia*, *Faecalibacterium*, *Roseburia*, and *Megamonas* [30]. Multiple studies consistently report a higher relative abundance of *Blautia* in the HD group compared to in healthy controls [14,29,31]. On the other hand, both Gao et al. and Koshida et al. highlighted increased levels of Erysipelotrichaceae [14,25]. Moreover, Chao et al. and Wu et al. observed elevated *Streptococcus* levels amongst HD patients [26,29]. Additionally, studies by Wu et al. and Zhang et al. found increased *Escherichia* and *Enterococcus*, but reduced *Bifidobacterium* in HD patients compared to in healthy individuals [26,32]. Please refer to Table 1 for a summary on the relative abundance of gut microbiota in HD patients compared to in healthy controls. 

The diversity and richness of intestinal flora in PD patients are considerably lower than in healthy people [28,33,34]. Li et al. identified *Blautia*, *Bacilli*, *Lactobacillales*, and *Ruminococcus gnavus* as the predominant flora in the PD group [28]. Another study by Tian et al. highlighted different predominant genera, including *Bacteroides*, *Faecalibacterium*, *Escherichia-Shigella* and Lachnospiraceae [35]. Studies comparing PD patients to healthy controls revealed mixed findings in regard to microbial abundance. Peng et al. and Wu et al. found increased *Bacteroides* abundance, whereas Li et al. reported decreased levels [27,28,33]. Additionally, both Peng et al. and Li et al. observed increased *Escherichia* levels in PD patients compared to in healthy individuals [28,33]. Please refer to Table 2 for a summary on the relative abundance of gut microbiota in PD patients compared to in healthy controls.

Compared to ESKD patients not receiving PD, PD patients showed a significant reduction in lactic acid-producing bacteria like *Bifidobacterium*, SCFA-producing bacteria like *Butyricicoccus*, and digestion-resistant starch bacteria like *Ruminococcus* [34]. However, Luo et al. found no significant effects of PD on the gut microbiota of ESKD patients [36]. 

The mode of KRT influences the composition and function of the gut microbiome. Whether PD or HD patients have a more favourable microbiota profile is debated, with differences often linked to metabolic and clinical variables. Two studies have underscored more pronounced gut microbiota alterations in HD patients compared to those undergoing PD [31,36]. Stadlbauer et al. found no difference in α-diversity between HD and PD patients, but reported distinctive genus-level abundances, in that PD patients exhibited higher levels of Thalassospira, *Eisenbergiella*, Ruminococcaceae, and *Coprococcus*, whilst HD patients showed elevated levels of *Escherichia-Shigella*, *Streptococcus*, *Enterobacter*, and *Blautia* [31]. The same study also identified reduced levels of *Faecalibacterium prausnizii* in HD and, to a lesser extent, PD [31]. Luo et al. observed increased *Blautia* and *Dorea* and decreased *Prevotella* in both PD and HD patients compared to in healthy controls, with HD patients showing significantly lower *Bacteroides* levels compared to PD patients [36]. Conversely, Hu et al. reported significantly lower α- and β-diversities in PD patients, indicating more severe intestinal flora disruption in PD compared to HD. The PD group also displayed decreased *Bacteroides* and increased Proteobacteria levels, along with notable changes at the family level, such as decreased Bifidobacteriaceae and Prevotellaceae, and increased Enterobacteriaceae and Enterococcaceae [37].

## 3. Factors Influencing Alterations in Gut Microbiota

**Figure 1:**
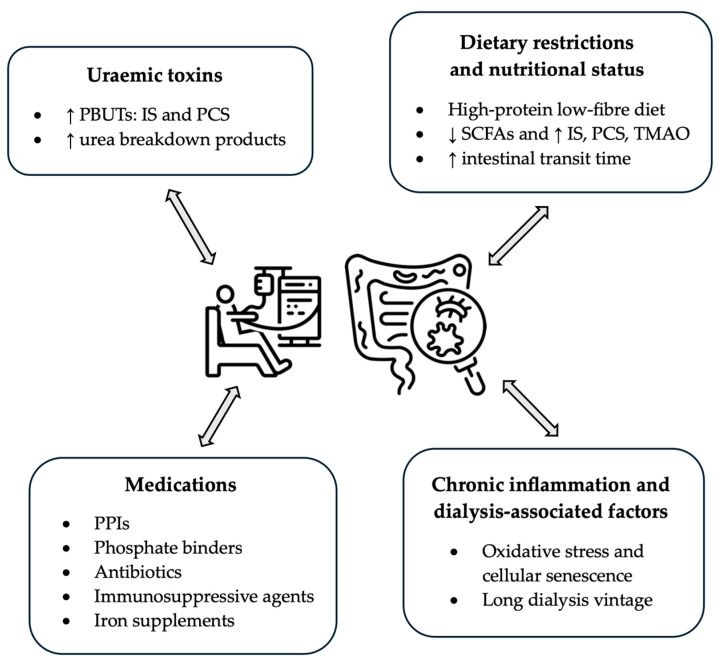
Key factors that may explain gut dysbiosis in patients receiving dialysis. PBUT: protein-bound uraemic toxin; IS: indoxyl sulfate; PCS: P-cresyl sulfate; SCFAs: short-chain fatty acids; TMAO: trimethylamine N-oxide; PPI: proton pump inhibitor.

Figure 1 summarises the key factors that may explain gut dysbiosis in patients receiving dialysis.

### 3.1. Uraemic Toxins

The accumulation of uraemic toxins is a hallmark of CKD, significantly accelerating disease progression, particularly in patients with ESKD undergoing dialysis treatment. Protein-bound uraemic toxins (PBUTs), such as indoxyl sulfate (IS) and p-cresyl sulfate (PCS), play crucial pathophysiological roles in CKD by inducing various cardiovascular and systemic complications. Whilst removing these toxins from the blood through dialytic techniques is a proven method to mitigate ESKD-related complications, conventional dialysis primarily targets the elimination of water-soluble compounds of low and middle molecular weight. Unfortunately, PBTUs are strongly protein-bound, rendering them less efficiently removed by standard dialysis methods [38,39]. Beyond the effects of CKD and incomplete dialytic removal of PBTUs, numerous studies have demonstrated that gut dysbiosis further exacerbates uraemic toxin generation [15,25,30].

In fact, the communication between gut dysbiosis and kidney function is bidirectional, as uraemia itself can upset the delicate balance of gut microbiota. High urea secretion into the intestinal lumen is hydrolysed by bacterial urease, forming ammonia and other toxic products, such as ammonium hydroxide. These substances damage the intestinal mucosa by degrading tight junction proteins, lowering pH, and increasing oxygen concentration, disrupting the environment for commensal bacteria [5,40,41]. Since normal intestinal microbiota predominantly consists of obligate anaerobes, which are sensitive to such changes, the symbiotic relationship between the microbiota and the host is compromised, resulting in dysbiosis [42,43]. 

Another plausible explanation is that impaired protein assimilation in uraemia may contribute to malnutrition, leading to an enhanced influx of undigested proteins into the distal intestine. This, combined with greater bioavailability of amino acids and peptides from malabsorption and/or intestinal oedema, creates an environment that stimulates the proliferation of proteolytic bacteria, thereby escalating the production of uraemic toxins [11,44].

### 3.2. Dietary Restrictions and Nutritional Status

The National Kidney Foundation recommends that non-dialysis-dependent CKD patients adhere to a diet low in protein, sodium, potassium, and phosphate, but high in fibre [45]. In contrast, dialysis patients are generally advised to consume more high-protein foods, such as meat, fish, poultry, fresh pork, and eggs. This recommendation stems from the fact that protein is lost during each dialysis session, placing this population at a high risk of malnutrition [46]. 

Many fibre sources, such as fruits, vegetables, and legumes, are high in potassium and phosphate, making it challenging to incorporate adequate fibre into a renal diet. The restricted intake of high-soluble fibre foods contributes to gut dysbiosis by enhancing proteolytic fermentation and decreasing saccharolytic fermentation. Notably, saccharolytic bacteria encompass *Bifidobacteria*, *Lactobacilli*, *Eubacteria*, *Bacteroides*, and *Prevotella* [40]. This shift diminishes the production of SCFAs, primarily acetate, propionate, and butyrate. SCFAs normally provide energy to the intestinal flora and allow amino acids that reach the colon to be incorporated into bacterial proteins and excreted, rather than being fermented into uraemic solutes. Moreover, SCFAs serve as substrates for the intestinal mucosa, supporting its functionality and integrity [11,47,48].

Proteolytic bacteria, on the other hand, produce toxic metabolites such as IS, PCS, and trimethylamine N-oxide (TMAO). Whilst TMAO is removed during dialysis, IS and PCS bind to albumin, thereby increasing their concentrations in plasma. Fibre intake lowers serum urea levels by facilitating faecal excretion of accumulated nitrogenous waste. Consequently, a low-fibre diet shortens intestinal transit time, prolongs amino acid fermentation, and exacerbates the imbalance in microflora composition, resulting in heightened production of undesirable solutes [11,47,48]. Similarly, the consumption of cheese and yoghurt is often restricted in CKD patients, due to their high phosphate content. These dietary restrictions not only limit the intake of foods with prebiotic and probiotic properties, but also contribute to reduced gut motility. Together, these factors promote gut dysbiosis and altered metabolic profiles in CKD patients [49]. 

In contrast to HD, PD allows for more liberal dietary restrictions due to its daily treatment sessions. Despite this distinction, malnutrition remains common amongst dialysis patients due to inadequate dietary intake and nutritional loss during dialysis [50]. Globally, malnutrition affects a significant portion of dialysis patients, with prevalence rates ranging between 18% and 75% [35,51].

Research by Lin et al. revealed significantly lowered α-diversity in HD patients experiencing protein energy wasting (PEW) [52]. Particularly notable was the marked reduction in the relative abundance of *Faecalibacterium prausnitzii*, a butyrate-producing bacterium [52]. Modifications in gut microbiota during periods of food deprivation may represent an adaptive response to optimise nutrient utilisation in low-calorie diets. This relationship operates in both directions, as dysbiosis resulting from inadequate dietary intake and lack of fibre may substantially contribute to the development of PEW [11]. 

### 3.3. Medications

Patients with ESKD are a complex cohort burdened with multiple comorbidities, often subjected to polypharmacy that includes proton pump inhibitors (PPIs), phosphate binders, prolonged courses of antibiotics, immunosuppressive agents, and iron therapy, amongst other medications [7,25,34,53]. Whilst essential for managing their condition, these medications can profoundly impair the gut microbiota. 

A study by Lin et al. found that HD patients treated with H2 antagonists (H2As) and PPIs exhibited a higher microbial dysbiosis index compared to the control group. H2A users showed a significant rise in the genera *Provetella 2*, *Phascolarctobacterium*, *Christensenellaceae* R-7 group, and *Eubacterium oxidoreducens* group, whilst PPI users had elevated levels of *Streptococcus* and *Veillonella*. Notably, PPI users demonstrated a more pronounced enrichment in *Streptococcus* compared to H2 receptor antagonist users [54]. Phosphate binders bind not only to harmful molecules such as p-cresol, endotoxin, advanced glycation end products, bile acids, and oxalates, but also to beneficial molecules like vitamin K, folic acid, and SCFAs, thus altering nutrient absorption and gut microbiota composition [11]. 

The use of antibiotics can disrupt the gut microbiota by eliminating both pathogenic and beneficial bacteria, leading to reduced microbial diversity, loss of various taxa, and the proliferation of antibiotic-resistant bacterial strains. This increases susceptibility to antibiotic-resistant infections and compromises the epithelial barrier [11]. Immunosuppressive treatments, such as corticosteroids, tacrolimus, cyclosporin, and mycophenolate mofetil (MMF), also impact the composition of gut microbiota. These agents operate through different mechanisms: glucocorticoids affect colonic mucus synthesis; tacrolimus enhances gut permeability; cyclosporin synergises with other immunosuppressants; and MMF alters the gut barrier. There exists a reciprocal effect between immunosuppressive drugs and the microbiota, as gut dysbiosis can influence the metabolism of these drugs [55]. 

Oral iron medications contribute to dysbiosis by suppressing SCFA-forming bacteria and promoting the growth of proteolytic bacteria [11]. Liu et al.’s study observed significant changes in species composition and diversity of gut microflora following both oral and intravenous iron therapies in HD patients. However, oral iron was remarkably more detrimental to intestinal bacteria compared to intravenous iron, as it significantly reduced α-diversity and the abundance of SCFA-producing bacteria in HD patients [56].

### 3.4. Inflammation and Immune Dysfunction

Chronic inflammation is commonly observed in CKD and dialysis patients. Whilst the precise mechanisms underlying the development of chronic inflammation in CKD are not fully elucidated, the aetiology of inflammation in CKD is usually multifactorial, involving numerous interacting factors in a uraemic environment. These factors include exogenous elements, such as dialysis membranes and central venous catheters; cellular contributors, like oxidative stress and cellular senescence; tissue-related issues, such as hypoxia, fluid overload, and sodium overload; microbial factors, including immune dysfunction and gut dysbiosis; and the retention of uraemic toxins, which stimulate the influx of inflammatory leukocytes [11,57,58].

A study by Li et al. found that elevated serum levels of IS in continuous ambulatory peritoneal dialysis (CAPD) patients correlated with increased leukocyte counts in abdominal dialysis fluid, suggesting that IS may be a risk factor for abdominal inflammation [28]. Whilst numerous studies have proposed that gut dysbiosis contributes to systemic inflammation [25,59], there is a consensus that chronic inflammation may also shape gut dysbiosis, thereby reinforcing the disease state [53,57,60,61].

Tight junctions primarily regulate the permeability of the intestinal epithelium. The process of inflammation disrupts these tight junctions, leading to increased gut permeability and intensifying dysbiosis. Dysbiosis, in turn, amplifies chronic low-grade inflammation, creating a vicious circle [62,63]. Thus, understanding and addressing these interconnected factors are crucial in managing chronic inflammation in CKD and dialysis patients.

### 3.5. Dialysis Modality, Vintage, and Frequency

As highlighted in previous studies, microbiome composition in ESKD patients varies significantly between dialysis modalities. Compared to pre-dialysis patients, alterations in the gut microbiota of HD patients are more pronounced than those in PD patients [31,36]. The distinct effects of these two dialysis modalities can be elucidated as follows. 

Firstly, the reduction in visceral blood flow under the compensatory mechanism of HD to maintain haemodynamic stability during ultrafiltration may cause intestinal hypoperfusion, recurrent regional ischaemia, and systemic circulatory stress. This disrupts the intestinal barriers and elevates the risk of bacterial translocation [64]. Previous studies have demonstrated that hepato-splanchnic blood flow substantially decreases during HD due to active splanchnic vasoconstriction, thus weakening the gut barrier. 

Secondly, HD removes uraemic toxins intermittently, whereas PD operates continuously [31]. 

Thirdly, gastrointestinal microbleeds induced by systemic anticoagulation therapies during HD treatments, combined with uraemic platelet dysfunction, can impair the structures and functions of the gut epithelial barrier. 

Finally, diet plays a crucial role in regulating the composition and metabolic activity of human gut microbiota. HD patients adhere to more stringent dietary restrictions compared to PD patients [36]. 

Despite differences in the frequency of sessions between HD and PD, prior research indicates that augmenting the duration and frequency of dialysis sessions improves the clearance of azotemic solutes that are loosely bound to proteins. However, intensifying dialysis frequency may compromise residual kidney function, thereby potentially diminishing overall solute clearance [65]. 

A study by Jiang et al. revealed that prolonged dialysis vintage, high peritoneal glucose exposure, and the loss of residual kidney function significantly affects gut microbiota homeostasis in PD patients, with dialysis duration being the most influential clinical factor [66]. Residual kidney function declines over time on PD. Since dialysis cannot entirely replicate kidney function, uraemic solutes and fluids accumulate in the gastrointestinal tract, contributing to gut flora alterations. Furthermore, as dialysis duration extends, the PD dose is increased, and hypertonic solutions are employed more frequently to enhance solute clearance and ultrafiltration. This results in the intestines being increasingly exposed to high levels of low-pH, hypertonic, lactate, and glucose-containing dialysate, which may aggravate gut dysbiosis [66]. Conversely, a study by Li et al. indicated that PD patients on dialysis for more than 60 months exhibited a favourable trend in flora composition, suggesting that the intestinal microecology of PD patients possesses a remarkable ability for self-regulation and remodelling [28].

## 4. Consequences of Alterations in Gut Microbiota

Alterations in gut microbiota have profound consequences, affecting various physiological systems, with significant metabolic implications being a primary concern. Over 150 uremic toxins, including IS and PCS, have been identified. These toxins are by-products of the intestinal microbial metabolism of tyrosine, phenylalanine, and tryptophan, and they progressively accumulate in CKD patients, involving gut *Escherichia-Shigella* [10,15,30].

High serum levels of IS and PCS in CKD patients negatively impact multiple organs and systems. Elevated IS levels are associated with increased oxidative stress on the vascular endothelium, reduced vascular elasticity, vascular smooth muscle proliferation, aortic calcification, and higher cardiovascular-related and overall mortality [67,68,69]. Chao et al. proposed that the production of PCS and IS increases the risk of vascular calcification, whilst Rossi et al. found a correlation between increased IS and PCS levels and elevated pro-inflammatory biomarkers, such as interleukin-6 and glutathione peroxidase, in CKD patients [29,70].

Furthermore, Lin et al. reported that IS increases the risk of peripheral vascular disease and vascular access thrombosis in CKD patients [71]. Mozar et al. and Barreto et al. found that IS suppresses osteoclast activity, leading to adynamic bone disease [68,72]. Nii-Kono et al. suggest that IS induces oxidative stress in osteoblasts, promoting resistance to parathormone and reducing bone formation [73]. Additionally, Lin et al. established a positive association between high IS levels and fibroblast growth factor 23 (FGF-23) [74]. IS has also been shown to have a significant profibrotic effect on the myocardium, stimulate myocyte hypertrophy, and increase the risk of atrial fibrillation [75]. Finally, there is a correlation between IS levels and the development of anaemic syndrome in CKD patients, as IS inhibits erythropoiesis, suppresses erythropoietin activity, and promotes the programmed cell death of erythrocytes [76,77]. 

SCFAs, such as acetate, butyrate, and propionate, are crucial products of the bacterial fermentation of plant-derived carbohydrates, primarily by the Bacteroidetes and Firmicutes phyla [49]. These SCFAs are essential for providing energy to colonic epithelial cells and maintaining gut barrier integrity by regulating tight junction proteins and modulating immune responses [49]. In CKD, gut dysbiosis leads to a reduction in SCFA production, which impairs intestinal barrier integrity and exacerbates CKD symptoms [78,79,80]. The compromised gut barrier allows toxic products to enter systemic circulation, establishing the “leaky gut” phenomenon as a key pathological mechanism in the gut–kidney–heart axis in CKD [81]. 

TMAO, a uremic toxin derived from the dietary intake of animal-derived choline and L-carnitine, as well as plant-derived betaine, is implicated in kidney and heart damage [82]. Elevated circulating levels of TMAO activate various pathways, increasing the expression levels of inflammasomes and cell enhancers, which can lead to vascular inflammation [83]. Inflammation is a critical factor in the progression of CKD, and TMAO has been shown to play significant roles in cardiovascular disease, atherosclerosis, and neurological disorders [84,85,86]. In the PD cohort, patients with higher TMAO concentrations have been observed to have a higher risk of developing new-onset peritonitis [87,88]. 

In a systematic review and meta-analysis by Li et al., which included 21 studies involving 15,637 patients, TMAO concentrations were associated with an increased risk of death in non-dialysis and non-Black dialysis patients [82]. TMAO can directly enhance platelet reactivity, thereby increasing the risk of thrombosis, including heart attack and stroke [89]. Additionally, animal studies have demonstrated that inhibiting TMAO production can delay disease progression in CKD mice, suggesting that TMAO may be a pathogenic factor in the progression of CKD [90].

Previous studies have demonstrated that metabolites related to cellular energy undergo significant alterations in patients undergoing dialysis. These changes are linked to diminished physical function and adverse outcomes, including increased mortality [91]. In a comprehensive metabolomics study, Zhu et al. found that elevated levels of kynurenic acid and S-adenosylhomocysteine, alongside reduced levels of L-glutamine, are associated with heightened cardiovascular risk in both HD and PD patients, compared to those with pre-dialysis stage 5 CKD [92]. Furthermore, increased concentrations of 2-keto-D-gluconic acid and C-reactive protein were indicative of greater risk for infective complications in both HD and PD patients [92]. 

## 5. Strategies to Modulate Gut Microbiota in Dialysis Patients

Modulating gut microbiota offers a promising approach to improving health outcomes in dialysis patients through dietary interventions and the use of probiotics and prebiotics. These strategies can restore microbial balance, enhance gut barrier integrity, and reduce systemic inflammation, thereby mitigating complications associated with CKD and ESKD.

Probiotics, defined as “live microorganisms that, when administered in adequate amounts, confer a health benefit on the host”, have been reported to influence blood urea nitrogen (BUN) levels [93]. Some studies indicate that probiotic supplementation decreases BUN levels [94,95], whilst others have reported increased BUN levels in HD patients following probiotic supplementation [96]. Taki et al. and Eidi et al. demonstrated that uremic toxins, such as PCS, decrease following probiotic administration [97,98]. However, a meta-analysis by McFarlane et al. did not show significant effects on uremic toxins from probiotic supplementation [99].

Research on the effects of probiotics on inflammation biomarkers has also yielded conflicting outcomes [100,101]. A more recent meta-analysis by Nguyen et al. in 2021 demonstrated a significant reduction in PCS levels in the HD population following probiotic supplementation [102]. Nonetheless, long-term studies are required, as short-term probiotic supplementation does not appear to influence plasma TMAO levels in HD patients [103]. 

Prebiotics, non-digestible food ingredients such as dietary fibres, various oligo- and polysaccharides, and resistant starches found naturally in many fruits, cereals, and vegetables have also shown benefits in modulating gut microbiota [96,104]. The fermentation process of prebiotics stimulates the growth of beneficial colonic bacteria, such as Lactobacilli species [1]. Studies have demonstrated that prebiotics reduce BUN, uremic toxins, oxidative stress, and inflammation [105,106,107,108]. Based on these findings, it is suggested that CKD patients should be encouraged to increase their intake of prebiotics due to their favourable effects [96]. 

Synbiotics, combined prebiotics and probiotics, are thought to have superior health-promoting effects compared to the isolated use of either prebiotics or probiotics [96,104]. Studies have shown that synbiotic supplementation alters intestinal microbiota, reduces BUN, delays the decline of the glomerular filtration rate (GFR) in non-dialysis CKD patients, and reduces circulating levels of uremic toxins [109,110,111,112]. Although lacking strong evidence, some studies suggest that bioactive compounds, such as curcumin and cranberry extract, can modulate gut microbiota by promoting the colonisation of beneficial bacteria and reducing oxidative stress and inflammation [96,113,114]. 

Other dietary modifications, such as adopting a low-protein diet, have also proven beneficial for non-dialysis CKD patients by inducing favourable changes in gut microbiota [115]. High-protein diets have been shown to increase circulating TMAO levels in healthy older males, whereas low-protein diets have resulted in lower TMAO levels in non-dialysis CKD patients [116,117]. Kandouz et al. and Patel et al. confirmed that higher protein intake increases the production of uremic toxins, with vegetarian HD patients exhibiting lower levels of uremic toxins compared to omnivorous HD patients [118,119]. Supplementation with oat β-glucan has been demonstrated to be safe and effective in reducing TMAO levels in CKD patients [120]. Adherence to a Mediterranean diet has also shown beneficial outcomes, with higher levels of SCFAs and lower levels of *E. coli* in stool samples [121,122].

## 6. Future Research

Whilst significant progress has been made in understanding gut microbiota alterations in CKD and ESKD patients, research specifically focusing on dialysis patients remains limited. Most studies have concentrated on the broader CKD and ESKD populations, often overlooking the unique microbiota changes and associated metabolic consequences in patients undergoing HD or PD. This gap in research leaves several unanswered questions about the specific interactions between dialysis modalities and gut microbiota, as well as the resultant impacts on patient outcomes. Understanding these interactions is crucial, given that dialysis patients experience distinct physiological and biochemical challenges that can further influence gut dysbiosis and its systemic effects.

Future research should prioritise longitudinal studies that monitor gut microbiota changes over time in dialysis patients, aiming to identify specific microbial markers associated with improved or worsened clinical outcomes. Investigating the effects of different dialysis modalities on gut microbiota composition and function can provide insights into optimising dialysis practices to mitigate dysbiosis. Additionally, exploring the roles of personalised nutritional interventions, including targeted probiotic, prebiotic, and synbiotic therapies, can offer tailored strategies to enhance gut health in this vulnerable population. Integrating advanced multi-omics approaches, such as metagenomics, metabolomics, and proteomics, will further deepen our understanding of the complex interactions within the gut microbiota and their systemic effects, ultimately guiding the development of novel therapeutic strategies for dialysis patients.

## 7. Conclusions

In summary, alterations in gut microbiota among dialysis patients are influenced by a myriad of factors, including diet, medication use, and the dialysis modality itself. These changes lead to significant consequences, such as increased production of uremic toxins, disruption of the intestinal barrier, and heightened systemic inflammation, all of which contribute to further kidney damage and its associated complications. The reviewed studies highlight the profound impact of gut dysbiosis on patient outcomes, underscoring the need for targeted research in this specific population to better understand and address these alterations. Given the critical role of the gut microbiota in influencing health outcomes, it is essential to incorporate strategies for modulating gut microbiota into the clinical management of dialysis patients. By prioritising these research directions and clinical practices, we can develop targeted interventions that have the potential to significantly improve health and quality of life for dialysis patients.

## Figures and Tables

**Table 1:** Relative abundance of gut microbiota in HD patients compared to in healthy controls.

Study	Gut Microbiota	Relative Abundance
Gao et al. [14]	*Blautia* ^g^ *Erysipelatoclostridium* ^g^ *Phascolarc-tobacterium* ^g^ *Sellimonas* ^g^ *Hungatella* ^g^ *Stenotrophomonas* ^g^	↑
	*Roseburia* ^g^	↓
Koshida et al. [25]	*Clostridium innocuum*^g^ *Porphyromonas*^g^ *Christensenellaceae_R_7*^g^ Streotococcaceae^f^ Lactobaccilales^o^ Bacilli^c^	↑
Wu et al. [26]	*Escherichia* ^g^ *Streptococcus* ^g^ *Lactobacillus* ^g^ *Enterococcus* ^g^ *Staphylococcus* ^g^ *Klebsiella* ^g^	↑
	*Bifidobacterium* ^g^ *Prevotella* ^g^ *Bacteroides* ^g^	↓
Chao et al. [29]	*Blautia* ^g^ *Streptococcus* ^g^ *Lachnospiraceae_unclassified* ^g^ *Ruminococcus_gnavus_group* ^g^ *Collinsella* ^g^ *Burkholderia-Paraburkholderia* ^g^	↑
Stadlbauer et al. [31]	*Blautia obeum* ^s^ *Clostridium citroniae* ^s^ *Clostridium bolteae* ^s^	↑
	*Faecalibacterium prausnizii* ^s^ *Roseburia intestinalis* ^s^ *Clostridium nexile* ^s^	↓
Zhang et al. [32]	*Escherichia coli* ^s^ *Enterococcus* ^g^	↑
	*Bifidobacterium* ^g^ *Lactobacillus* ^g^	↓

^c^: class; ^o^: order; ^f^: family; ^g^: genus; ^s^: species; ↑: increase; ↓: decrease. HD: haemodialysis.

**Table 2:** Relative abundance of gut microbiota in PD patients compared to in healthy controls.

Study	Gut Microbiota	Relative Abundance
Yasuno et al. [20]	*Lactobacillus* ^g^	↓
Wu et al. [27]	Bacteroidetes^p^	↑
	Firmicutes^p^	↓
Li et al. [28]	Proteobacteria^p^ Enterobacteriaceae^f^ *Escherichia-Shigella*^g^ *Blautia*^g^	↑
	Bacteroidetes^p^ *Faecalibacterium*^g^	↓
Stadlbauer et al. [31]	*Clostridium citroniae* ^s^ *Clostridium bolteae* ^s^	↑
Peng et al. [33]	*Bacteroides thetaiotaomicron* ^s^ *Bacteroides fragilis* ^s^ *Escherichia coli* ^s^ *Parabacteroides unclassified* ^s^ *Ruminococcus gnavus* ^s^	↑

^p^: phylum; ^f^: family; ^g^: genus; ^s^: species; ↑: increase; ↓: decrease. PD: peritoneal dialysis.

## Data Availability

Not applicable.
